# Listen and You Shall Hear: Lessons from Theology and Pastoral Care

**DOI:** 10.5334/pme.2052

**Published:** 2026-03-25

**Authors:** Lavjay Butani

**Affiliations:** 1 University of California Davis Medical Center, CA, USA

## Abstract

Medicine is inherently a spiritual profession; patients and health care professionals both seek meaning and connections beyond just themselves, especially in the context of crises. Yet, the spiritual aspects of care have fallen to the wayside, with the commercialization of the business of health care. In this brief perspective, I integrate lessons from theology and pastoral care into a busy clinical and educational setting to help reclaim the moral dimensions of the care giving relationship.

The pediatric community celebrates the legacy of Drs. Steven Miller and Richard Sarkin [1] each October by highlighting the value of humanism that they promoted, a quality that is at the heart of medicine, and one that is challenged in the context of economic pressures that prioritize throughput and efficiency. Clinicians and trainees can strengthen the provision and role modeling of humanistic care by cultivating and demonstrating a mindful presence and listening skills, alongside their technical expertise. In this viewpoint, using concepts from pastoral care and theology, we provide practical strategies on how a clinician and a trainee, in the midst of the busyness of their lives, can pause mindfully and be present to listen to the most important stakeholder in the room- the patient (from the latin *patiens:* ‘those who experience suffering’). We do so, explicating on the care giving encounter in four phases, and by providing a teachable framework for nonverbal skills, the EMPATHY framework [2], that we have operationalized in the context of a teaching environment, providing examples of specific behaviors and feedback skills that can be used by a clinician during the visit. Only through care that is compassionate and responsive can we reclaim the service mission of medicine, improving patient satisfaction [3] and also reducing physician burnout [4].

Clinicians and trainees need to be consciously aware of the inherent power dynamics in the caregiving relationship, to monitor them and keep them in balance. Like providers of pastoral care, healthcare providers must use a combination of agential and receptive power [5]. Agential power is the power that health care professionals have by virtue of the competencies they acquire through training. However, all too often, we overuse agential power at the expense of receptive power—the power that arises from being completely present to receive the stories of those who seek care. Care conversations must be ‘like a dance, where dancers alternate in taking the lead and following the other,’ balancing the skills of listening with technical expertise [5]. Borrowing further from the field of theology, the care-giving experience must balance preparation and planning. Unlike traditional ‘planned’ theology, which emphasizes an omnipotent, static God with a preordained plan, process theology posits a changing, persuasive God who co-creates with free-willed entities. Similarly, clinicians and trainees must strive to resist overreliance on rigid frameworks (checklists) and strictly predetermined agendas (akin to planned theology) and be flexible and open to co-creation, as they encounter the human mystery that awaits upon entering a patient’s room. While attention to checklists and other scaffolds that help standardize care and improve patient safety are of utmost importance, this must not occur at the expense of exploring and addressing what patients need. This willingness to listen and hear what care-seekers truly need as opposed to our perceptions of their needs, allowing them to set the agenda, gives back some control to the ‘humanistic expert’- patients themselves (process theology). In other words, while we must prepare and plan before entering the room, we must also be wary of establishing a pre-determined outcome, bringing not only our knowledge but also our professional habits and dispositions to the caregiving experience. In the next section, we demonstrate strategies, which can be used at various stages of the patient-healthcare professional-trainee interaction: before entering the ‘room’, during the care visit, leaving the room, and after the care visit. We introduce each of these moments with portions of a poem; acts of intentionality such as the recitation of these poems can serve as rituals that slow us down and remind us of the human dimensions of care.

**Before entering the ‘room’**

‘Mindfully walking into the hospital
I smile to all I meet
Dedicating my compassionate energy
For the transformation of suffering’

‘Mindful of my body
I breathe in to relax my body
I breathe out to relax my body
To bring a calm presence to those in need’ [6]

In the midst of busy schedules, we can sometimes lose sight of the spiritual nature of our caregiving profession. Before entering a patient’s room or walking into a hospital, consciously reminding ourselves of the moral nature of our profession and our commitment to serve can center us. Mindful practices intentionally slow us down and increase our presence; such practices are akin to rituals that connect us with ourselves, others, the moment, and the transcendent [7]. In a time constrained context, rituals can take the form of a simple act of intentionality, such as washing one’s hands slowly before entering the room, touching one’s stethoscope and reminding oneself of the Hippocratic oath, resting one’s hands on the door handle leading to the patient’s room for a brief moment, slowing our pace as we walk towards the patient’s room, sauntering (a slow deliberate walk) from the work room towards the exam room, or reciting aloud a ‘gatha’ (short poem) such as the one shown above. These micro-acts of intentionality serve the function of rituals, albeit in a secular context, and are accessible to all, irrespective of the spiritual and religious beliefs of the stakeholders.

In less time-limited settings, or when one is working with the same trainee over a longer period of time, we can engage the trainee in creating learning goals, which include at least some aspects of humanism. These goals or mission statements can be made into a visual reminder (team charter) [8] that can be posted and referred to, if not every day, at least in challenging moments. Any such activity should be done with intentionality and with the goal of preparing to receive the patient as a person, with curiosity and humility, much as we might a guest at our home. While a comprehensive discussion of the ethics of consent is beyond the scope of this perspective, it would be remiss to not highlight the primacy of patient agency, especially in the context of a teaching environment. While uncommonly discussed in the literature, it is essential that all care-giving encounters with patients, especially children, be grounded on individualized trainee readiness based on competency, and on creating an environment that empowers patient/families to choose whether, and to what extent, to participate in the trainee teaching experience [9].

And then asking for permission to enter, we can cross the threshold.

**During the visit**

‘Mindful of my mind
I search for an agenda
Finding an agenda, I drop it
I open, like a flower, to what the patient needs’ [6]

Mindful practitioners facilitate the creation of an environment where the patient is always recognized as an expert. Mindful practice entails not only presence (physical and mental), but also humility, curiosity, and flexibility in going where patients lead us. While much has been discussed in the literature pertaining to verbal communication that can promote a humanistic relationship [2], less emphasis has been given to nonverbal strategies that are so impactful in relationship building. During the caregiving visit, the EMPATHY framework (adapted in Table 1), is one framework that can be used during teaching encounters, through role modeling (‘activated demonstration’) [10], and in giving feedback to trainees, demonstrating how to channel ‘receptive power’ in the care-giving encounter.

**Table 1:** EMPATHY [2] (non-verbal practices that prioritize receptive power).


APPROACH TO HUMANISTIC CARE DURING THE VISIT	WHAT IT MEANS IN A BUSY CLINICAL PRACTICE	HOW A PRECEPTOR CAN EXPLICITLY ROLE MODEL BEFORE THE ENCOUNTER	HOW A PRECEPTOR CAN GIVE FEEDBACK TO A TRAINEE AFTER THE ENCOUNTER

**E**ye Contact	Establishing and maintaining eye contact with the patient	‘I position myself in the room such that I can maintain eye contact with the patient at all times and take notes only during pauses in the conversation.’	‘I noticed that you were typing notes when the patient’s mother frowned at a question you asked. Maintaining eye contact during the interview will allow you to notice non-verbal cues that may be worth addressing.’

**M**uscles of facial expression	Keeping an open and relaxed face; subtle mirroring of patient expressions; nodding in affirmation; head tilting; furrowing of the brow to express concern, smiling to convey understanding/support	‘I have found that mirroring emotions in the room, such as sadness and joy through my own facial expressions can demonstrate empathy.’	‘When the patient’s father questioned your recommendation to start antibiotics, I saw that your face tensed up. I am concerned that this may have affected your rapport building. What are your thoughts?’

**P**osture	Sitting down to the level of the patient during verbal interactions; keeping arms uncrossed and relaxed; torso facing patient	‘I am cognizant of how I position my arms as I talk to people. Crossing my arms across my chest has been mistaken in the past to be a sign that I am closed off from patients.’	‘I really appreciated how you got down onto the floor of the exam room and examined the patient as she was playing. Towering over children when we interact with them can be a scary experience for them.’

**A**ffect	Noticing patient affective cues and responding to them in a way that explores and validates expressed feelings	‘When I sense strong emotions in the room, I try to name what I am sensing and ask for clarity to ensure that I am not misreading affective cues.’	‘Your humility in noticing and asking the patient’s mother if her tears were an expression of fear, sadness, or some other emotion, was laudable. It really opened up the healing process.’

**T**one of voice	Using a gentle, low pitched and unpressured voice	‘I have received feedback that my slow and unpressured voice is calming to patients and so I make it a point to be consciously aware of how I am speaking.’	‘Did you notice that you were talking extremely fast during the patient visit? Let us talk more about that.’

**H**earing the whole patient	Aligning (‘coupling’) verbal and non-verbal responses to what the patient is conveying as a whole [11]	‘In my experience patients’ facial expressions can often be misinterpreted. I have made it a practice now to mindfully observe patients’ body language in the context of their verbal communication, and even then, I practice humility and confirm if my understanding is accurate.’	‘I noticed that when the patient’s mother laughed, you smiled in response. I wonder, though, if her laughter was an expression of nervousness since we had just shared with them the uncertainty of her child’s medical diagnosis. What are your thoughts?’

**Y**our response	Practicing curiosity about one’s own responses (physical and affective) to the interaction using reflective practice [12]	‘That was a challenging encounter for me, and I found myself getting irritated. I would really like to hear your thoughts on how you saw things during the visit.’	‘Let’s talk about what went well for you from an affective domain and what was a challenge during the last visit.’

**Leaving the room:**

‘Mindful of being hospitable
I remember I am also their guest
I greet with open arms and
I leave with palms together (as in, with a mind of prayerfulness and
gratitude)’ [6]

As we prepare to leave the room, expressions of gratitude directed towards patients convey that the health care team recognizes the vulnerability of those who suffer and seek care. This vulnerability is even more apparent when there is a trainee in the room. Demonstrations of humility, respect, and reverence towards those we are called to serve can help strengthen the therapeutic relationship for ongoing healing.

**After the care visit:** Taking an opportunity to reflect and debrief with the trainee about what they noticed during the encounter, and both giving and receiving feedback, can promote the trainee’s professional identity formation and our own resilience. Debriefing with the trainee on the human dimensions of care enables them to appreciate the value we place on the sacrosanct experience of caring for another.

In summary, presence and listening skills, balanced with our technical expertise, are essential in the provision of effective, appropriate, and holistic care to patients. Role modeling these skills, teaching them to our trainee, and giving them feedback on their humanistic behaviors, can empower us to bring humanism front and center into our professional practices.
